# Metabolomics in Radiation-Induced Biological Dosimetry: A Mini-Review and a Polyamine Study

**DOI:** 10.3390/biom8020034

**Published:** 2018-05-29

**Authors:** Changhyun Roh

**Affiliations:** 1 Biotechnology Research Division, Advanced Radiation Technology Institute (ARTI), Korea Atomic Energy Research Institute (KAERI), 29, Geumgu-gil, Jeongeup-si, Jeonbuk 56212, Korea; chroh@kaeri.re.kr; Tel.: +82-63-570-3133; 2 Radiation Biotechnology and Applied Radioisotope Science, University of Science Technology (UST), 217 Gajeong-ro, Daejeon 34113, Korea

**Keywords:** ionizing radiation, polyamines, metabolomics, radiation biodosimetry, biomarker

## Abstract

In this study, we elucidate that polyamine metabolite is a powerful biomarker to study post-radiation changes. Metabolomics in radiation biodosimetry, the application of a metabolomics analysis to the field of radiobiology, promises to increase the understanding of biological responses by ionizing radiation (IR). Radiation exposure triggers a complex network of molecular and cellular responses that impacts metabolic processes and alters the levels of metabolites. Such metabolites have potential as biomarkers for radiation dosimetry. Among metabolites, polyamine is one of many potential biomarkers to estimate radiation response. In addition, this review provides an opportunity for the understanding of a radiation metabolomics in biodosimetry and a polyamine case study.

## 1. Introduction

Globally, there are concerns over the elucidation risks associated with radiation exposure; hence, it is important to comprehend the biological effects of radiation exposure. Driven by the need to detect the presence of radiation exposure, a biomarker to monitor potentially exposed situation after radiological accidents can be developed and would be extremely valuable for biological response. Ionizing radiation (IR) has inevitably become major public health concerns due to exposure from artificial sources such as radiological medical usage and from natural sources like space travel [1,2]. Especially, radiation exposures from nuclear and radiation accidents and the threat of terrorism including use of radioactive isotopes are big issues [2,3]. The interaction of radiation with a biological system is a common factor irrespective of the condition of accidental radiation exposure. This involves deposition of energy to cellular targets either directly or involving highly reactive free radicals. DNA, RNA, proteins, lipids, carbohydrates and many metabolites may lead to various pathological conditions [4,5,6,7,8,9]. The monitoring of damage by radiation has been of great interest for developing simple and reliable methods for detection of radiation exposure as an essential factor in clinic. Cytogenetic analysis, particularly the dicentrics chromosome aberration assay of peripheral blood lymphocytes is a gold standard technique for estimating the extent of radiation exposure [6,7,8,9]. Despite considerable advances in detection methods, it has several drawbacks such as being time-consuming and labor-intensive. To overcome this bottleneck, a metabolomics platform is introduced as a methodology for high-throughput assessment of the radiation dose received.

Metabolomics is a rapidly advancing field that aims to characterize the concentration changes of all small molecules existing in a sample. Furthermore, application of metabolomics technologies to the understanding of physiology, toxicology, and disease progression has led to appreciable advances by defining novel drug and carcinogen metabolites, as well as biomarkers of disease. At the same time, metabolomics technologies have contributed to a general understanding of how metabolites and their concentrations change under defined conditions. However, in contrast to transcriptomics and proteomics, broad-based metabolomics studies have not been used to analyze the cellular effects of IR. Radiation metabolomics, the application of a metabolomics in biodosimetry analysis to the field of radiobiology, give a promise to increase the understanding of biological responses to ionizing radiation [10,11,12,13,14,15]. Radiation exposure triggers a complex network of molecular and cellular responses that impact metabolic processes and alter the levels of metabolites. Such metabolites have potential as biomarkers for radiation dosimetry. An understanding of the molecular and cellular effects of ionizing radiation have depended on profiling technologies such as genomics, transcriptomics, and proteomic platforms. Such efforts have shown ionization radiation-induced perturbations of DNA, RNA, and protein molecules and have been successful in developing biomarkers that provide information regarding radiation-induced phenomena such as the threshold dose. Furthermore, integrating data from combinations of such platforms, in the spirit of emerging systems biology, has given investigators the ability to reconstruct and analyze ionization radiation responsive pathways. However, pathways generated from such analyses remain incomplete without similar global measurements of metabolites. Despite recent advances in metabolic profiling developments, changes in small-molecule metabolites remain underexplored and underexploited. This is particularly unfortunate because, as the end products of transcriptional and proteomic signaling events, metabolites may represent the most incisive and accurate indicators of the state of cellular physiology, toxicology, and disease progression, and have led to appreciable advances by defining novel drug and carcinogen metabolites, as well as biomarkers of disease. A biomarker, which means biological marker, is an important feature that can be used to measured or assess kinds of biological characteristics or parameters with different types of effects being induced by radiation responses [16]. Although viable technology is well established to analyze free radicals for mechanisms of DNA repair from radiation damage in biological models, its clinical and protection level of use for even humans is challenged with various factors [17,18]. Here, we review the current status of radiation metabolomics that have contributed to a general understanding of how metabolites and their biomarkers change like polyamines under defined conditions. This article provides an understanding of radiation metabolomics in biodosimetry and a polyamine case study.

## 2. Radiation Metabolomics in Biological Dosimetry

### 2.1. Metabolomics Platform in Biodosimetry

Exposure to ionizing radiation elicits a set of complex biological responses involving gene expression and protein turnover that ultimately manifest as dysregulation of metabolic processes representing the cellular phenotype. Metabolomics in radiation biodosimetry is one of the core disciplines for discovery of novel biomarkers. The discipline focuses on the study of low molecular weight metabolites in biological responses by ionization radiation. The quantitative complement of all metabolites is defined as the metabolomics and sample-specific metabolomics can be studied, e.g., biofluids (blood or urine) metabolomics or tissue metabolomics.

**Figure 1:**
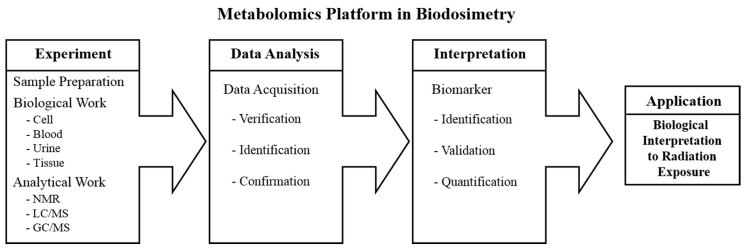
Workflow diagram involves the metabolomics approaches. Abbreviations: NMR, nuclear magnetic resonance; LC/MS, liquid chromatography/mass spectrometry; GC/MS, gas chromatography/mass spectrometry.

Radiation metabolomics operates with a workflow [19] starting from a biological question and experiment, proceeding through sample collection and preparation, analytical experiments to acquire data, data pre-processing and analysis followed by biological interpretation. This general process for conducting radiation metabolomics studies is displayed in the flow diagram in Figure 1.

The first step is sample generation from radiation effects. A well-planned radiation effect with multiple doses, multiple time points for sample collection and preparation are very important, and the analytical work is global profiling that usually involves an analysis for identification of spectra of nuclear magnetic resonance (NMR) or mass spectrometry (MS) spectral data including gas chromatography (GC) and liquid chromatography (LC) to determine new biomarkers or spectral patterns of biomarkers that can be related to radiation exposure. The second step involves data acquisition may be undertaken. Principal component analysis (PCA) is applied to the NMR or MS data initially to look for patterns and outliers, and to determine if there are any easily discernable biomarkers. After PCA, many other types of supervised methods like partial least squares-discriminate analysis (PLS-DA) can be employed for further statistical data analysis. This is an exciting time to be working in the field of metabolomics because of advances on every front. Improvements are legion, from instrumentation for biomarker discovery, to computer methods for data analysis, to understanding of pathways, to instrumentation for targeted analysis. The recent published results, and ongoing studies in cell lines and animal models, indicate a dose response, and show clustering and discrimination based on combined detections of groups of metabolites derived from samples such as urine that can be obtained non-invasively. Data from these studies analyzed by bioinformatics methods led to the discovery-phase identification of groups of metabolites useful for screening and biodosimetry. This is extremely encouraging and makes extension of the work to human populations important, even with the limitations inherent in such a study. Finally, the power of the results from discovery is realized if they can be utilized to streamline targeted analysis and to provide new kinds of information in discovery. Ion mobility methods have simplified quantitative detection of biomarkers, but have also allowed the elimination or simplification of sample cleanup and pre-separation. With the reduction in overall analysis time within minutes, it is possible to follow kinetics or responses at a rate that is otherwise difficult, and it is possible to process data from larger cohorts in discovery. The third step is data interpretation for biomarkers. At this step, the biomarker to radiation exposure will be elucidated. The results provide putative biomarkers that were previously not reported or originate from a biological mechanism not known before. Once discovery studies have been performed, the putative biomarkers are validated in the general things where the biomarker will be applied to assess specificity and selectivity. This applies an analytical method for absolute quantification. This metabolomics method can then be employed for future application in radiation biology. The application of metabolomics to the radiation biodosimetry has led to the description of a number of metabolites that have the potential to be developed into robust biomarkers applied in biological interpretation to radiation exposure. In general, the metabolomics method provides clues that lead to the discovery of new and better biomarkers, as well as to insightful hypotheses regarding mechanisms of radiation effects.

### 2.2. Metabolomics Technologies

The role technological developments provided in scientific discoveries has been suggested that progress in science depends on new techniques, new discoveries and new ideas, probably in that order. Metabolomics suggests the simultaneous and relative quantification of thousands of different metabolites within a given sample using sensitive and specific methodologies such as LC or GC coupled to MS for assessment of biomarker discovery in response to radiation. The first definitions of metabolomics were reported in 1998 [20,21]. From that time, there have been huge advances in methodological and analytical technologies that have led to the discovery of biomarkers and greater knowledge regarding diseases. Technological advances from the 1960s with gas chromatography/mass spectrometry (GC/MS), liquid chromatography/mass spectrometry (LC/MS) [22] and NMR spectroscopy [23,24] allowed the first holistic studies of mammalian biofluids to be performed. In the last 15 years, technological advances have driven metabolomics to its current status. A combination of sample preparation and analytical platforms is recommended to acquire good coverage of detected metabolites. Today, metabolomics is a routinely applied tool with greater than 19,390 publications reported in PubMed (as of March 2018). Especially, the study of radiation metabolomics accounts for 450 publications reported in PubMed (as of March 2018).

### 2.3. Potential Biomarkers

**Figure 2:**
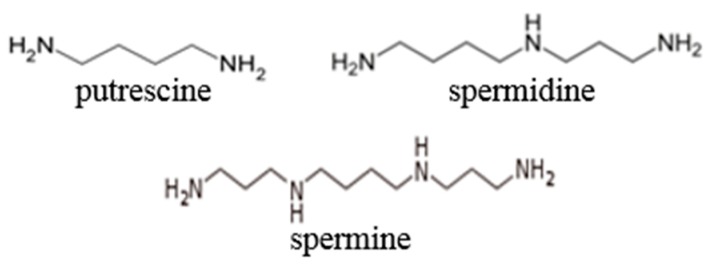
Chemical structure of polyamines.

Biomarkers are biological characteristics that are objectively measured and evaluated as indicators of biological responses to radiation exposure. The search for biomarkers of effective dose and the early effects of ionization radiation exposure in both humans and experimental animals has a history spanning several decades. Biomarkers that can help identify exposed individuals are critically important in the event of mass casualty incidents. Metabolomics approaches for biomarkers allow the simultaneous, quantitative analysis of thousands of different metabolites within a given sample [25]. It aims at identifying unique fingerprints of specific disturbances such as example effects of exposure to radiation. Polyamines (putrescine, spermidine, and spermine) are aliphatic polycations present in all cells, where they have pleiotropic effects that allow their linkage to DNA, RNA, and proteins, for example, with a relevant regulatory role in a number of steps of cell metabolism. The putrescine, spermidine, and spermine from polyamines play a pivotal role in living organisms [26,27,28]. Polyamines are low molecular weight organic polycations displaying their important biological activities having mediated a multitude of in vivo processes. In general, the polycationic nature of polyamines is important for their biological activities and their in vivo interaction with multiple kinds of molecules such as polyanionic nucleic acids and proteins. The chemical structures are presented in Figure 2.

**Figure 3:**
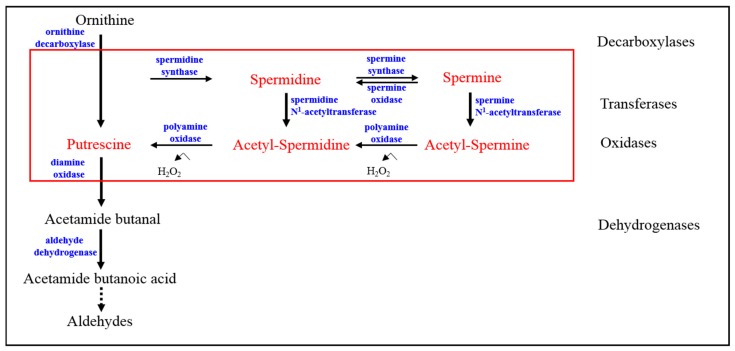
Potential biomarkers in polyamine metabolism.

Regarding their functions, polyamines play a critical role in cell metabolism [29], cell proliferation [30], and cell differentiation [31] through cellular processes including the metabolic pathways of synthesis, degradation and transport [32,33]. Especially, spermidine from polyamine is an aliphatic polycation that is present in all cells, where it has pleiotropic effects that allow their linkage to DNA, RNA, and proteins. Spermidine plays a regulatory role in a number of steps of cell metabolism in living organisms. The pathway of polyamine metabolism including metabolites and genes is represented in Figure 3. Since polyamine metabolism is involved in many biologically relevant processes, polyamines and most of the enzymes involved in their metabolism have been extensively studied. All of them have been isolated, purified, and kinetically characterized and their genes have been cloned. Much information is available concerning the enzymes, their activities, regulation, functional features and gene expression [34]. Polyamines are produced from the metabolism of cationic amino acids of arginine. The first step in their biosynthesis is a decarboxylation. Their terminal catabolism by amine oxidases releases compounds with a high potential to produce damage: aldehydic compounds that eventually can be exported or oxidized and reactive oxygen species. Transferases play a role in both metabolic pathways. Finally, *S*-adenosylmethionine is a donor of aminopropyl groups in the biosynthesis of polyamines [35,36,37,38]. As for the polyamine pathway, the steady state intracellular concentrations of polyamines are influenced by the regulation of the key enzymes of their metabolic pathways, but also by the regulation of transporters for their uptake and release. On the other hand, the storage of very important quantities of amines into vesicles of their producing cells is a well-known fact for amines, including polyamines [39,40]. Increases in polyamine concentration have also been linked to carcinogenesis [41]. Recent reports have shown that adequate polyamine levels are required for the maintenance of cell proliferation and cell differentiation. Ionizing radiation generally results in increased oxidative stress triggered by the direct ionization of biological responses (e.g., DNA, protein), and indirect modifications to cellular components through reactive oxygen species generated by the radiolysis of water, such as hydroxyl radicals. Acute and chronic levels of radiation-induced oxidative stress have been demonstrated to cause deleterious cellular injury as measured by an increase in the extent of lipid peroxidation, DNA, and aromatic hydroxylation, as well as the upregulation of various antioxidant enzymes, which reflects an adaptive cellular response mechanism to radiation damage. A metabolomics platform in radiation biodosimetry is a powerful tool to study post-radiation changes in polyamine metabolism.

**Figure 4:**
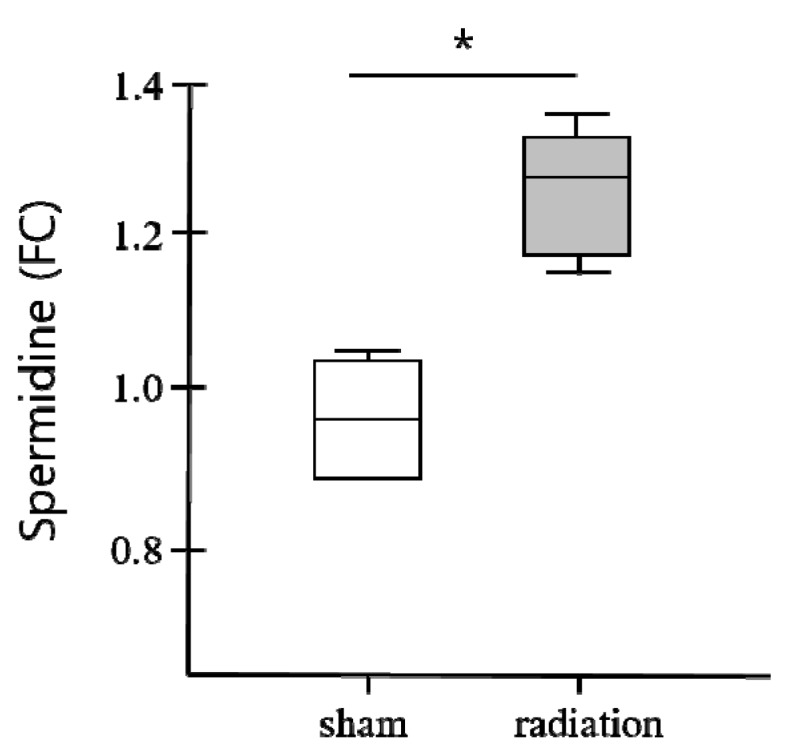
Changes in spermidine biomarker in response to gamma radiation of 6 Gy. *p* value was calculated by using one-way analysis of variance (ANOVA). * Difference with a *p* < 0.05 was considered as significant. Abbreviation: FC, fold change [42].

The behavior of spermidine kinds of polyamines as biomarkers was investigated by the Roh group in a mouse model exposed to an acute whole-body sublethal dose of 6 Gy. A time lag of 12 h post-irradiation showed spermidine as a significantly elevated metabolite among sham and γ-irradiated mice [42]. As shown in Figure 4, the plot showing serum-normalized fold change (FC) for the spermidine biomarker was calculated with respect to the average of the metabolite in the sham-irradiated group. Another work used UPLC-ESI-TOF-MS (ultra performance liquid chromatography coupled with electrospray ionization-time of flight-mass spectrometry) coupled with PCA for the analysis of serum from mice exposed to 3 Gy of radiation showed changes of DNA damage biomarkers and a N1-acetylspermidine in polyamines. Thus, findings from this study emphasize the role of polyamine metabolism toward impacting the efficiency of DNA damage and repair which suggests that polyamine is a potent biomarker and a non-invasive feature for radiation responses [43,44]. It was also reported that high polyamine levels are required to protect healthy cells against reactive oxygen species (ROS)-triggered damage while polyamine confers on cancer cells a higher resistance to these oxidative attacks which indicates the protective effects of polyamines as free radical scavengers [45,46].

## 3. Conclusions

Metabolomics platforms use small molecules including endogenous metabolites and exogenous compounds obtained from nutrients, in numerous ways. Metabolites are used to provide energy, furnish building blocks to create cells and tissues, and can be used to signal at the cellular or physiological level. The importance of metabolites in radiation biodosimetry has driven the development of metabolomics approaches to enable the detection and quantification of biological metabolites from biological samples to radiation response. Changes in metabolite levels can reveal important information about the metabolites and metabolic pathways affiliated with a radiation response. Metabolites like polyamines appear to be a good biomarker to estimate radiation damage lethality or diseases in living organisms. The future of metabolomics in understanding radiation response is an exciting prospect. The result of this new understanding will be both a deeper knowledge of biological pathways to radiation exposure and affected systems, and new technologies that enhance discovery and provide clinical tools.
